# Correlation analysis between serum total IgE and FeNO and idiosyncratic reaction in bronchiolitis

**DOI:** 10.1016/j.clinsp.2024.100384

**Published:** 2024-05-15

**Authors:** XiaoYing Xu, WeiNing Han, WeiPing Han

**Affiliations:** a Department of Pediatrics, Dongying People's Hospital (Dongying Hospital of Shandong Provincial Hospital Group), Dongying City, Shandong Province, China; b Department of Pediatrics, Wudi People's Hospital, Binzhou City, Shandong Province, China

**Keywords:** Bronchiolitis, Total immunoglobulin e, Fractional exhaled nitric oxide, Idiosyncratic Reaction

## Abstract

**Objective:**

This article focused on the correlation between the changes of serum total Immunoglobulin E (IgE) and Fractional exhaled Nitric Oxide (FeNO) and idiosyncratic reactions in children with bronchiolitis.

**Methods:**

One hundred children with bronchiolitis and fifty healthy children were enrolled. Serum total IgE and FeNO were assessed, and the diagnostic value for bronchiolitis and the correlation with the severity of bronchiolitis were analyzed. Bronchiolitis children were divided into idiosyncratic + bronchiolitis and non-idiosyncratic + bronchiolitis groups, the relationship between serum total IgE and FeNO and idiosyncratic reaction was determined, and the diagnostic value of serum total IgE and FeNO for idiosyncratic bronchiolitis was examined.

**Results:**

FeNO in bronchiolitis children was lower than that in healthy children but there was no significant difference in serum total IgE levels between the two populations. Serum total IgE increased while FeNO decreased with the aggravation of bronchiolitis in bronchiolitis children. The serum total IgE was positively correlated while FeNO was negatively correlated with the severity of bronchiolitis. Serum total IgE was higher in children with idiosyncratic bronchiolitis, but serum total IgE and FeNO were not the risk factors for idiosyncratic bronchiolitis; Area Under the Curve (AUC) of serum total IgE and FeNO for the diagnosis of idiosyncratic bronchiolitis was less than 0.7.

**Conclusion:**

Serum total IgE and FeNO in children with bronchiolitis are related to disease severity and idiosyncratic reaction. FeNO has a diagnostic value for bronchiolitis, but not for idiosyncratic bronchiolitis.

## Highlights

• FeNO in bronchiolitis children was lower than that in healthy children.
• Serum total IgE increased while FeNO decreased with the aggravation of bronchiolitis in bronchiolitis children.
• Serum total IgE was higher in children with idiosyncratic bronchiolitis, but serum total IgEand FeNO were not the risk factors for idiosyncratic bronchiolitis.
• Area Under the Curve (AUC) of serum total IgE and FeNO for the diagnosis of idiosyncratic bronchiolitis was less than 0.7.

## Background

Bronchiolitis is one of the common lower respiratory tract infectious diseases that are common in children under 2 years old. The main manifestations are cough, shortness of breath, paroxysmal wheezing, wheeze sound and moist rales in both lungs. Previous studies have shown that children with a history of bronchiolitis are more likely to experience wheezing, decreased lung function, and airway hyperresponsiveness.^1^^,^^2^ For the pathogenesis of bronchiolitis, it is more that Th1 cell subsets are inhibited and Th2 cell subsets are dominant, which is very similar to the hyperthyroidism of Th2 in bronchial asthma advocated by many scholars.^3^^,^^4^ Fractional exhaled Nitric Oxide (FeNO) can be used as a clinical marker reflecting eosinophilic airway inflammation, and has a certain value in the diagnosis and disease assessment of asthma. However, related reports pointed out that the acute phase of bronchiolitis was mainly neutrophilic inflammation, and the chronic phase is a mixture of eosinophilic airway inflammation and neutrophilic airway inflammation,^5^^,^^6^ that is, FeNO is differentially expressed in different disease stages. Allergen-specific Immunoglobulin E (IgE) detection is the main clinical diagnostic method, and IgE exerts critically in chronic airway inflammation and hyperresponsiveness and activates inflammatory cells to release inflammatory cytokines.^7^^,^^8^ However, there have yet to be any reported studies that have investigated the association between the IgE and FeNO levels in patients with bronchiolitis. Based on this, the present study explored the correlation between the changes of serum total IgE and FeNO and idiosyncratic reaction in children with bronchiolitis, and provided evidence for the evaluation of the disease and the early judgment of idiosyncratic reaction.

## Materials and methods

### Clinical data

From March 2019 to October 2021, 100 children with bronchiolitis (bronchiolitis group) and 50 healthy children (healthy group) all obtained from Chinese Han patients were enrolled. There was no significant difference in the general data between two groups (all *p* > 0.05, Table 1). Inclusion criteria: 1) Meet the diagnostic criteria for bronchiolitis;^9^ 2) Be admitted to hospital within 24 h of onset; 3) Complete clinical data. Exclusion criteria: 1) Patients with a history of respiratory tract infection within the past 2 w; 2) Patients administrated with systemic inhaled glucocorticoids within 4 w; 3) Patients administrated with bronchodilators within 24 h; 4) Patients with allergic rhinitis; 5) Patients taken anti-allergic drugs in the past week; 6) Patients with combined immunodeficiency diseases.

**Table 1:** Comparison of general data.

Groups		Bronchiolitis group (*n* = 100)	Healthy group (*n* = 50)	χ2/t	p
Gender	Male	67	30	0.715	0.398
	Female	33	20		
Age		1.05 ± 0.21	1.01 ± 0.23	1.065	0.289
Gestational age		40.03 ± 0.15	39.97 ± 0.16	1.506	0.134
Height (cm)		73.24 ± 3.37	72.95 ± 3.58	0.487	0.627
Body weight (kg)		10.07 ± 2.14	10.13 ± 2.06	0.164	0.87
Allergy history in first degree relative		18	4	2.663	0.102
Immediate family smoking history		31	14	0.143	0.705
Immediate family history of lung disease		17	10	0.203	0.652

### Methods

#### Serum total IgE and FeNO

Eight milliliters of peripheral venous blood were collected from every child after 4 h of fasting. Total IgE was analyzed by ELISA (BD Biosciences, San Diego, CA, USA), and FeNO was by a NIOX FeNO analyzer (Aerocrine AB, Solna, Sweden) according to the FeNO standardization determination guidelines recommended by the American Thoracic Society/European Respiratory Society.

#### Idiosyncratic reaction determination

An Allergy Screen system (R-Biopharm AG, Darmstadt, Germany) was applied to detect allergen-specific IgE. If one or one abnormal allergen-specific IgE is positive, it can be determined as the idiosyncratic reaction.^10^

#### Severity of bronchiolitis

Bronchiolitis was graded into mild, moderate and severe (Table 2).

**Table 2:** Severity of bronchiolitis.

Item	Mild	Moderate	Severe
Feeding amount	normal	To half normal	To half normal or food refusal
Respiratory rate	Normal or slightly faster	> 60 breaths/min	> 70 breaths/min
Chest wall inspiratory tri-concave sign	Mild or no	Moderate (obvious rib gap depression)	Severe (very obvious rib gap depression)
Nose flaring or moaning	No	No	Ye s
Oxygen saturation	> 92 %	88 %∼92 %	< 88 %
Mental state	Normal	slight or intermittent irritability, irritability	Extreme irritability, sleepiness, coma

Note: Any item in the table can judge moderate to severe bronchiolitis.

### Observation indicators

(1) The serum total IgE and FeNO of the bronchiolitis group and the healthy group were compared, and their diagnostic value for bronchiolitis and their correlation with the severity of bronchiolitis were analyzed.
(2) According to the detection results of serum allergens, the bronchiolitis group was divided into idiosyncratic + bronchiolitis group and non-idiosyncratic + bronchiolitis group, and the relationship between serum total IgE and FeNO and the idiosyncratic reaction was analyzed, as well as the diagnostic value of IgE and FeNO for the idiosyncratic reaction.

### Statistical methods

SPSS 22.0 software was applied to process data, enumerate data were expressed in% and compared by χ^2^ test; measurement data were expressed by mean ± Standard Deviation (SD) after the normality test and compared by unpaired Student's *t*-test. The diagnostic value of serum total IgE and FeNO in bronchiolitis was analyzed by Receiver Operating Characteristic (ROC); the correlation between serum total IgE and FeNO and the severity of bronchiolitis was analyzed by Spearman test. The relationship between serum total IgE and FeNO and the idiosyncratic reaction was analyzed by logistic regression analysis; *p* < 0.05 suggested a statistical difference.

## Results

### Comparison of serum total IgE and FeNO

**Fig. 1:**
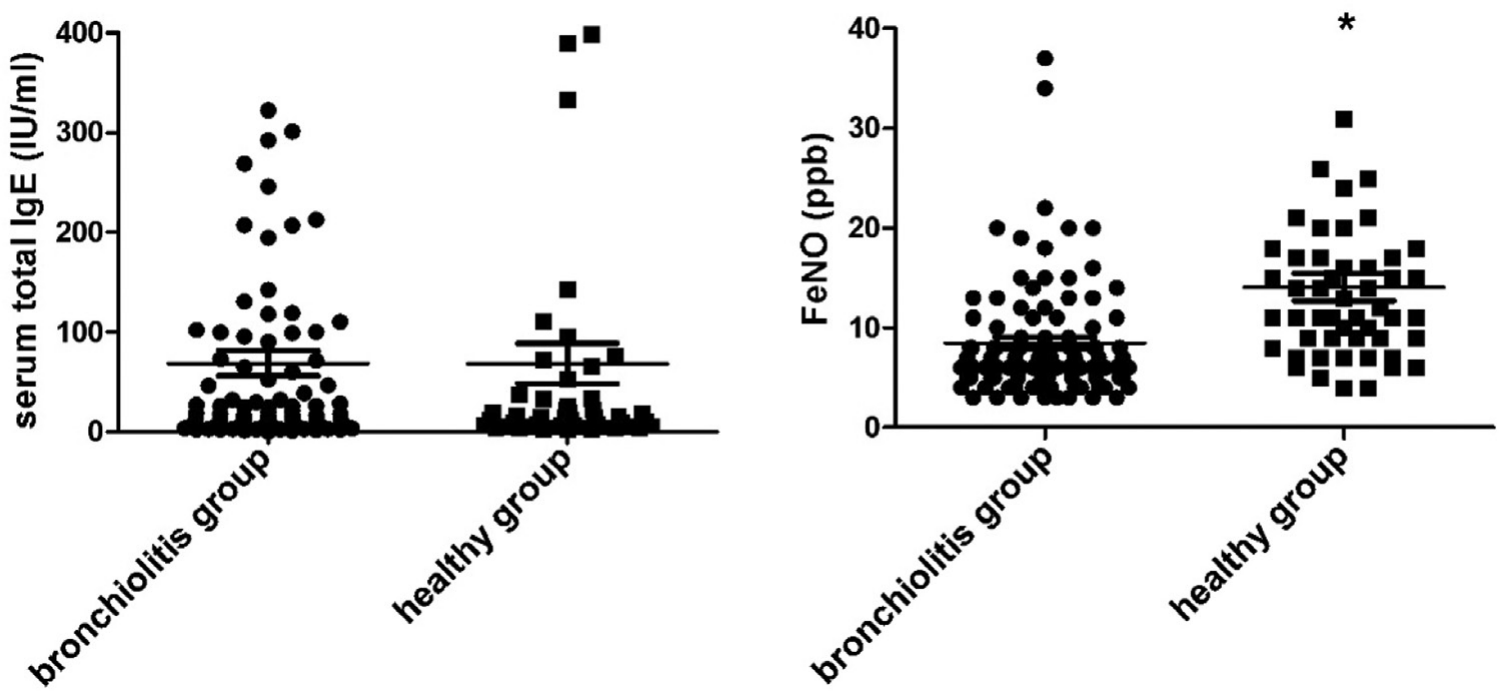
Serum total IgE and FeNO in bronchiolitis and healthy children. Note: The total FeNO was determined using the NIOX FeNO analyzer, whereas the serum total IgE level was measured using the ELISA; *p < 0.05compared with healthy children.

Bronchiolitis children expressed higher FeNO than healthy children (*p* < 0.05), but there was no difference in serum total IgE between the two groups (*p* > 0.05, Fig. 1).

### Diagnostic value of serum total IgE and FeNO for bronchiolitis

**Fig. 2:**
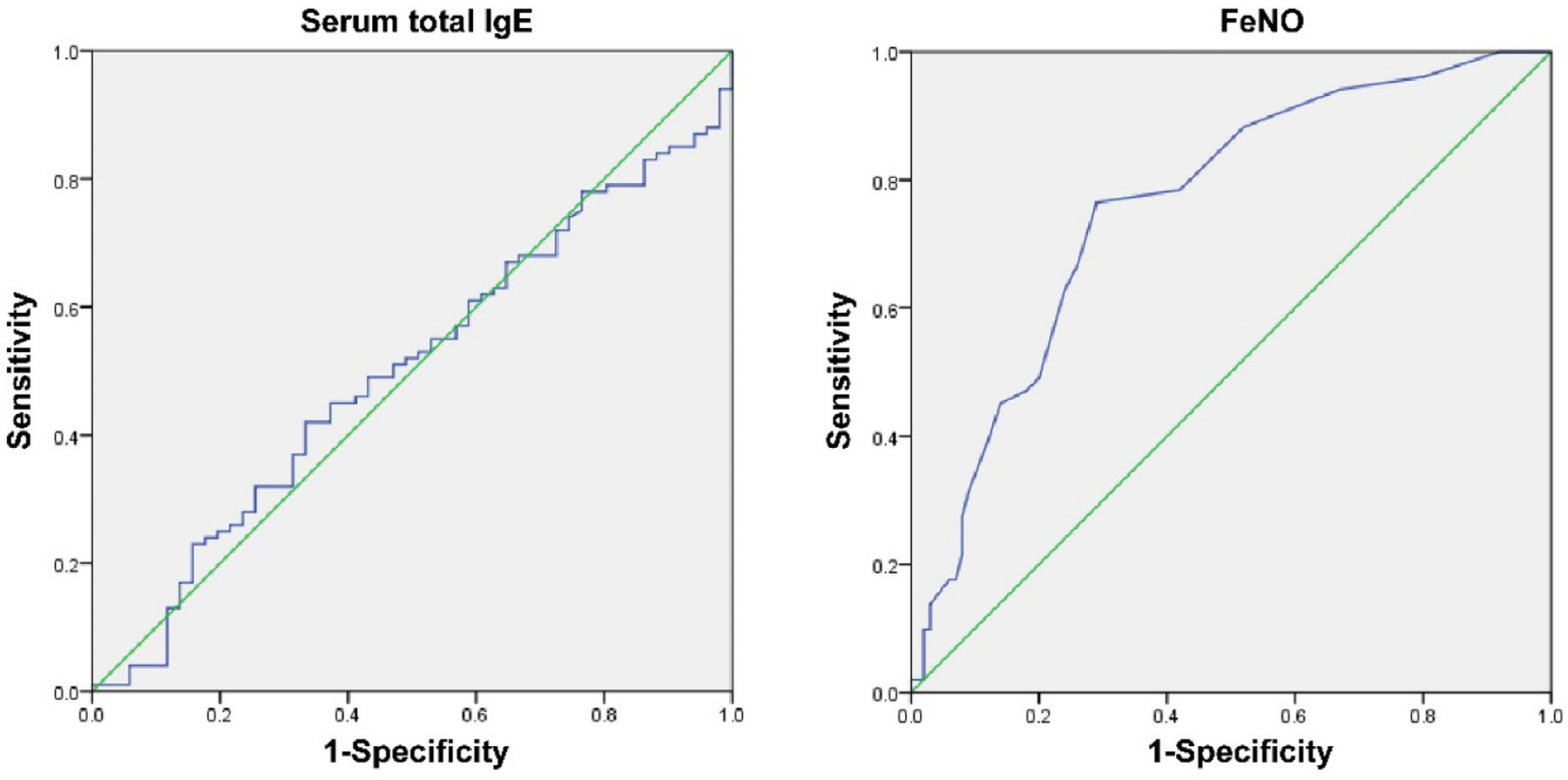
Receiver Operating Characteristic (ROC) curve of serum total IgE and FeNO in the diagnosis of bronchiolitis children. Note: AUC (95 % CI) for total IgE = 0.5 (0.992∼0.596), AUC for total FeNO = 0.759 (0.680∼0.838).

The Area Under the Curve (AUC) of serum total FeNO was greater than that of serum total IgE (*p* < 0.05, Table 3, Fig. 2).

**Table 3:** Diagnostic value of serum total IgE and FeNO for bronchiolitis.

	Cut-off value	AUC	SE	95 %CI	Specificity	Sensitivity
Serum total IgE	2.83	0.5	0.049	0.992∼0.596	98.04	12
FeNO	≤8	0.759^a^	0.04	0.680∼0.838	76.47	71

Note: Compared with serum total IgE.

a *p* < 0.05.

### Serum total IgE and FeNO in bronchiolitis of different severity

**Fig. 3:**
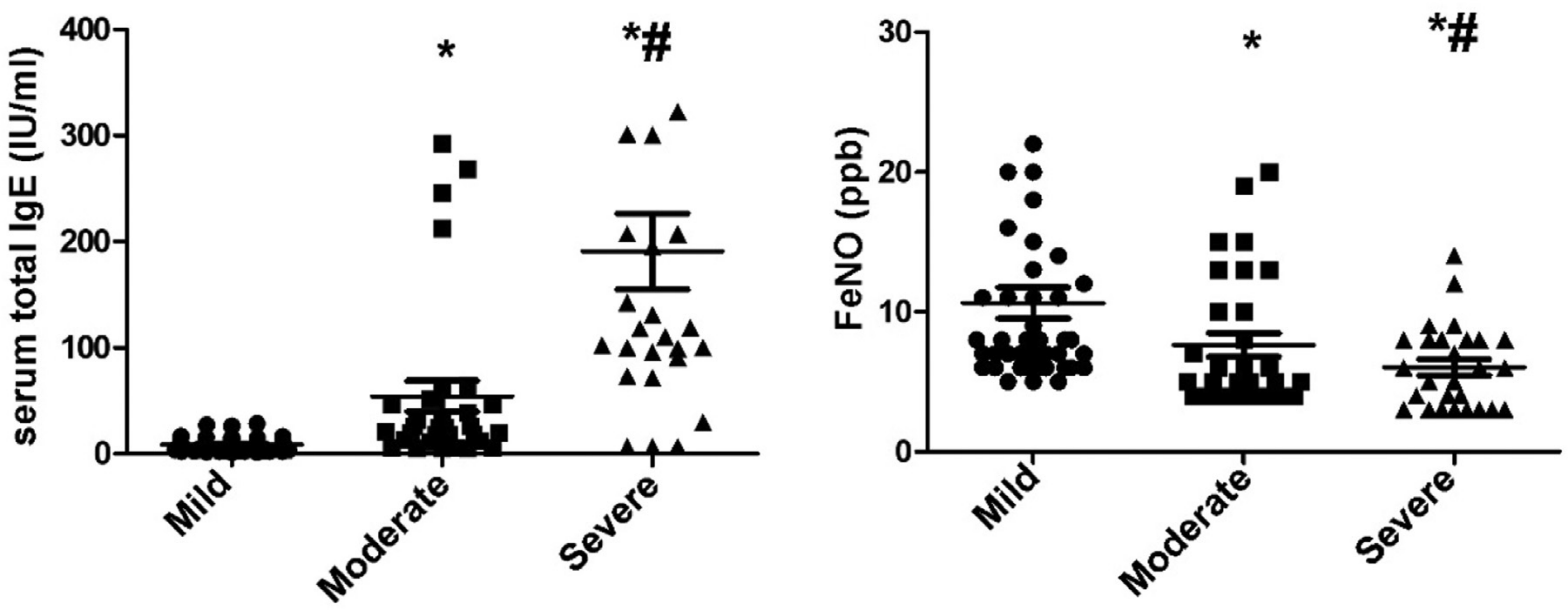
Serum total IgE and FeNO in children with mild, moderate, and severe bronchiolitis. Note: Serum total IgE showed an increasing trend while FeNO showed a downward trend with the aggravation of bronchiolitis; *p<0.05 compared with mild children; # Compared with moderate children.

Serum total IgE showed an increasing trend while FeNO showed a downward trend with the aggravation of bronchiolitis (*p* < 0.05, Fig. 3).

### Correlation analysis between serum total IgE, FeNO and the severity of bronchiolitis

**Fig. 4:**
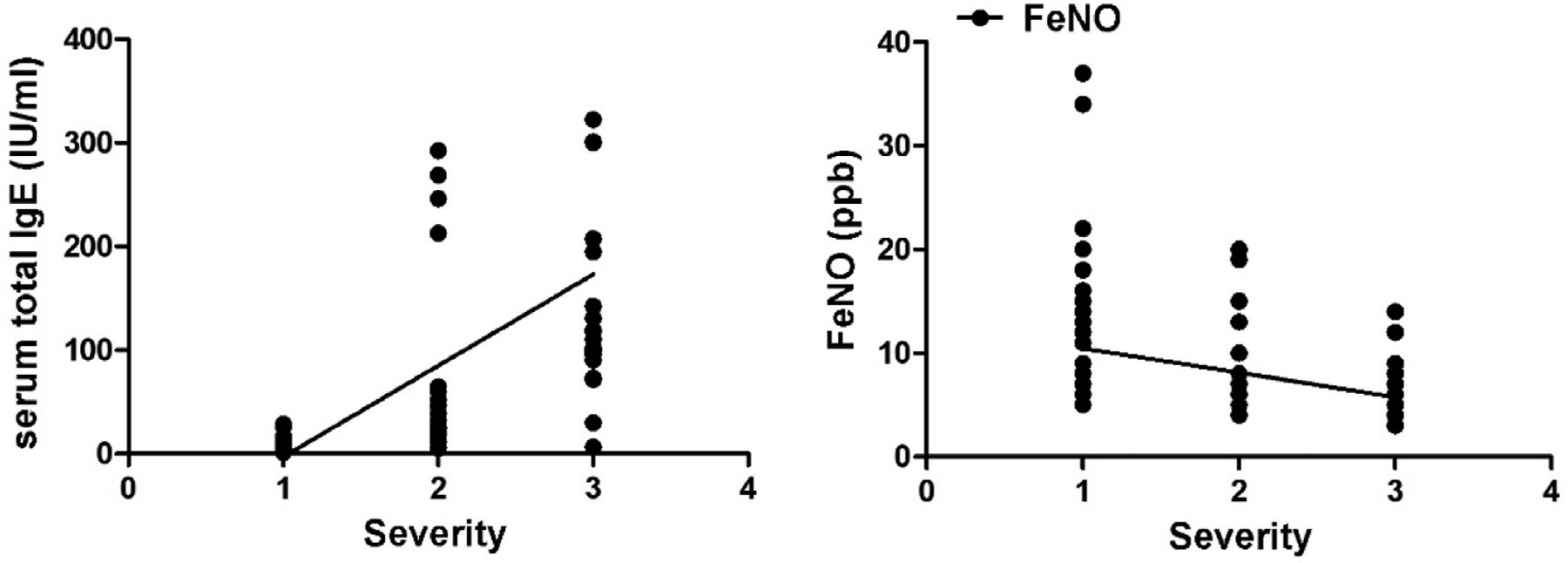
Correlation analysis of serum total IgE, FeNO and the severity of bronchiolitis. Note: The correlation between serum total IgE and FeNO and the severity of bronchiolitis was analyzed by Spearman test.

Serum total IgE was positively correlated while FeNO was negatively correlated with the severity of bronchiolitis (all *p* < 0.05, Fig. 4).

### Comparison of serum total IgE and FeNO in idiosyncratic and non-idiosyncratic bronchiolitis children

**Fig. 5:**
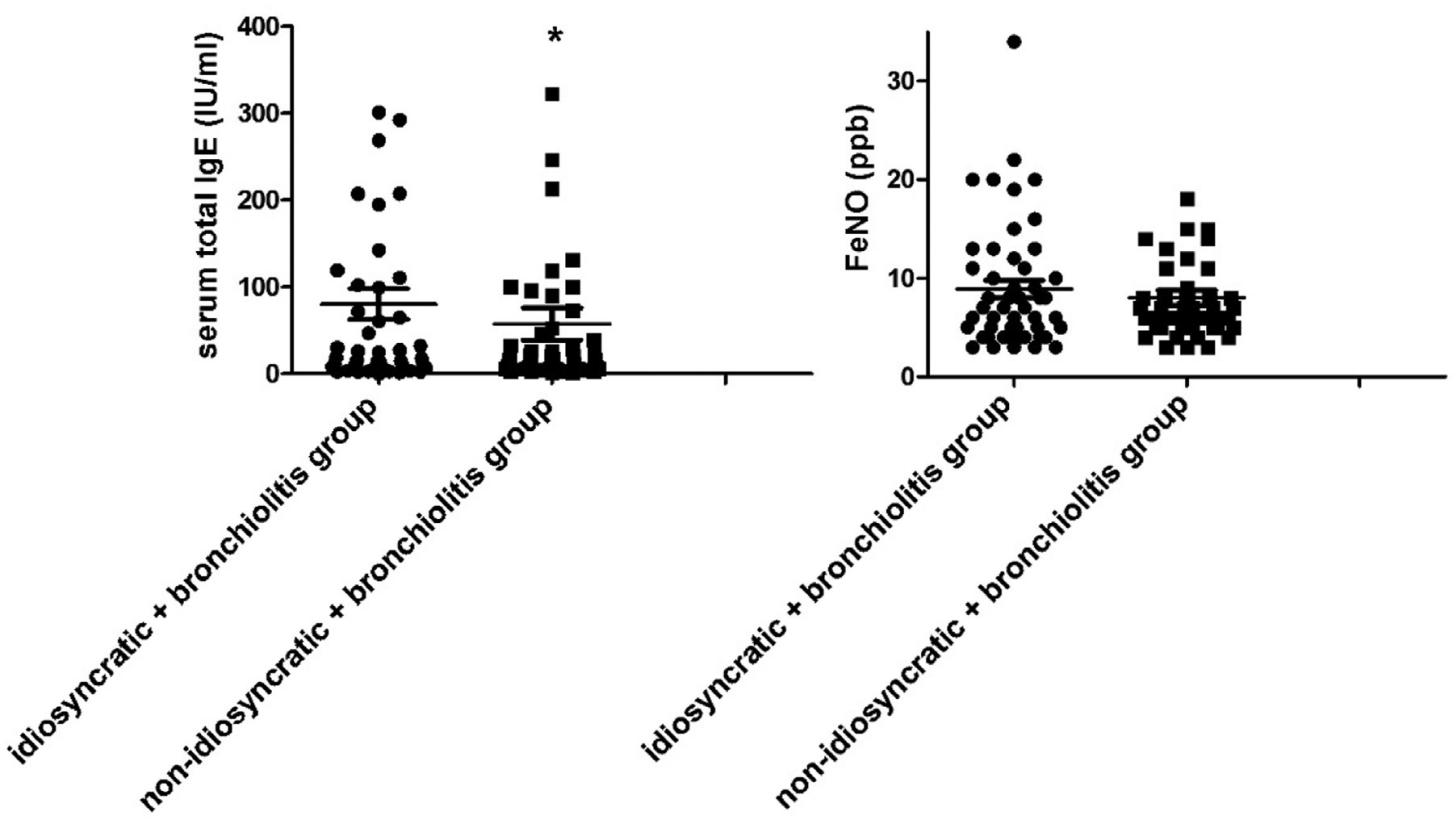
Diagnostic value of serum total IgE and FeNO for idiosyncratic bronchiolitis. Note: The bronchiolitis group was divided into idiosyncratic + bronchiolitis group and non-idiosyncratic + bronchiolitis group, and the relationship between serum total IgE and FeNO and idiosyncratic reaction was analyzed; *p < 0.05 compared with idiosyncratic + bronchiolitis group.

Only serum total IgE suggested a significant difference between idiosyncratic and non-idiosyncratic bronchiolitis children (*p* < 0.05), but FeNO showed no obvious change between idiosyncratic and non-idiosyncratic bronchiolitis children (*p* > 0.05, Fig. 5).

### Logistic regression analysis

The logistic regression analysis showed that Serum total IgE and FeNO were not the risk factors which affected the idiosyncratic reaction (*p*>0.05, Table 4).

**Table 4:** Logistic regression analysis of serum total IgE, FeNO and idiosyncrasy.

Indicators	β	SE	Wald χ2	OR	95 %CI	p
Serum total IgE	0.171	0.235	0.529	1.186	0.749∼1.881	0.467
FeNO	−0.026	0.036	0.522	0.974	0.908∼1.046	0.471
Constant	−0.32	0.377	0.72	0.726	0.347∼1.520	0.396

Assignment: Serum total IgE (≥ 2.29 IU/mL, < 2.29 IU/mL, 0); FeNO (≥ 8 ppb, < 8ppb, 0).

### Diagnostic value of serum total IgE and FeNO

**Fig. 6:**
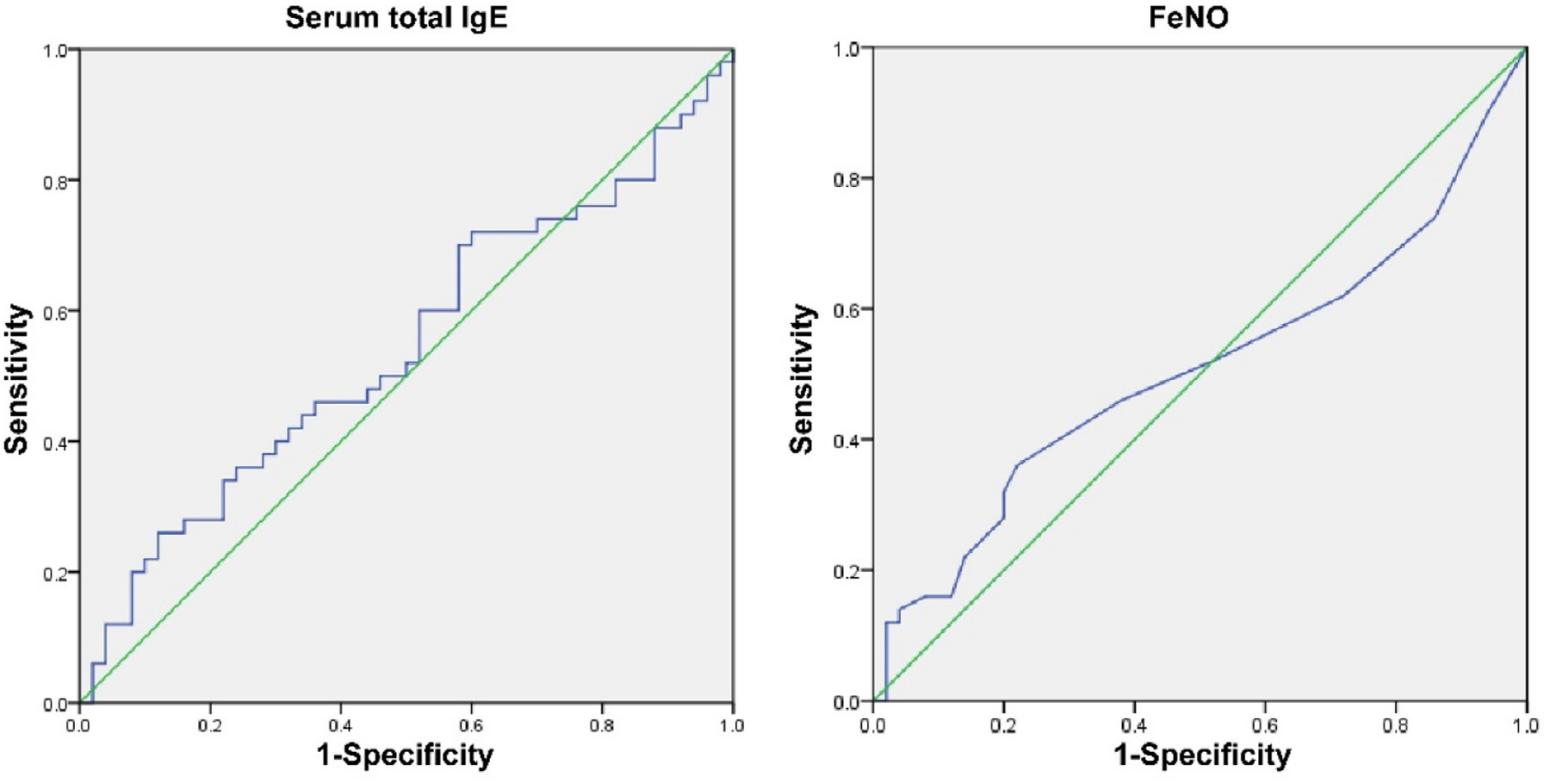
ROC curve of serum total IgE and FeNO for the diagnosis of idiosyncratic bronchiolitis. Note: AUC (95 % CI) for total IgE = 0.543 (0.440∼0.643), AUC for total FeNO = 0.059 (0.405∼0.608).

The AUC of serum total IgE and FeNO for the diagnosis of idiosyncratic bronchiolitis was less than 0.7 (Table 5, Fig. 6).

**Table 5:** Diagnostic value of serum total IgE and FeNO on idiosyncrasy.

Indicators	Cut-off value	AUC	SE	95 %CI	Specificity (%)	Sensitivity (%)
Serum total IgE	2.29 IU/mL	0.543	0.058	0.440∼0.643	88	26
FeNO	8 ppb	0.506	0.059	0.405∼0.608	78	36

## Discussion

Bronchiolitis is an infection-induced lower respiratory tract disease, which was mostly caused by the respiratory syncytial virus and other viral infections. The main pathological changes are epithelial cell necrosis, peripheral lymphocyte infiltration, submucosal congestion, edema, and increased glandular secretion.^11^^,^^12^ Cough, shortness of breath, and other symptoms can be significantly relieved in most patients after active anti-infection and related treatment, but some children experience recurrence and may develop asthma. Relevant studies have pointed out that FeNO levels in children with bronchiolitis will also change to varying degrees.^13^^,^^14^ In recent years, FeNO has been more and more widely used in respiratory diseases and is considered to be one of the markers to monitor airway inflammatory diseases.^14^ FeNO is a Th2 cell-mediated marker of airway inflammation. From the perspective of the pathogenesis of bronchiolitis, viral infection stimulates cells to secrete various cytokines and inflammatory mediators, which could induce immune responses and damage capillaries.^15^^,^^16^ The hyperfunction of Th2 cell subsets and the inhibition of Th1 cell subset functions are the main pathogenic mechanisms of bronchiolitis.^17^, ^18^, ^19^ When Th2 function is in a dominant position, the cytokines and inflammatory mediators secreted by Th2 will also increase, which can promote the differentiation, proliferation, and activation of B cells, produce IgE, and increase serum IgE, while IgE and basophils can participate in allergy, causing a large number of eosinophils to infiltrate locally. This article indicated that serum total IgE in bronchiolitis and healthy children was not significantly different, which is different from a previous study,^20^ which may be related to the small sample size or related to the fact that the children are in the acute phase and the immune status has not changed significantly. This study also found that serum total IgE was positively correlated with the severity of bronchiolitis, indicating that serum total IgE was related to the disease progression. FeNO levels in bronchiolitis children were lower than in healthy children, suggesting that eosinophilic inflammation is not dominant during the acute phase of bronchiolitis, but is dominated by neutrophilic inflammation.^21^ It was also determined that the AUC of FeNO for the diagnosis of bronchiolitis was greater than 0.7, indicating its diagnostic value for bronchiolitis.

Idiosyncrasy refers to a kind of special constitution related to heredity, and people with idiosyncrasy are prone to diseases such as bronchial asthma and allergic rhinitis. Changes in serum total IgE levels are related to bronchiolitis,^22^ suggesting that there may be abnormal serum total IgE levels in children with idiopathic reactions. As the authors know IgE is synthesized by B cells in the mucosal lymphoid tissue of the respiratory tract and digestive tract and can mediate allergic reactions. Changes in serum IgE levels in children with bronchiolitis are related to disease progression.^23^ IgE receptors can be expressed on the surface of cells such as eosinophils and basophils, and mediate the immediate allergic reactions and delayed allergic reactions. Children with idiopathic reactions are prone to allergic reactions, suggesting that there may be differences in serum total IgE levels between the idiopathic children and the non-idiopathic children. This paper showed that the total serum IgE in children with idiosyncratic bronchiolitis was higher than that in those with non-idiosyncratic bronchiolitis, indicating that if children with idiosyncratic bronchiolitis, serum total IgE levels may increase. FeNO level in idiosyncratic children is significantly higher than that in non-idiosyncratic children, regardless of whether they have asthma.^24^ This study showed that FeNO in idiopathic children was slightly higher than that in non-idiosyncratic children, but the difference between the two was not statistically significant, which was slightly different from the results of the above study. In children with acute bronchiolitis, the mediating effect of eosinophils in children with acute bronchiolitis is weak, resulting in the decrease of FeNO levels in the body. Further analysis found that serum total IgE and FeNO were not risk factors affecting idiosyncratic reaction, and the AUC for the diagnosis of idiopathic was less than 0.7, indicating that serum total IgE and FeNO had no diagnostic value for idiopathic bronchiolitis. However, the present study had one limitation. Due to resource limitations, the sample size of children in the study is small. Thus, investigation with more study children should be performed to draw more reliable conclusions from a wider range of sample data.

## Conclusions

To sum up, the changes in serum total IgE and FeNO in children with acute bronchiolitis are related to the severity of the disease and idiosyncratic reaction, and FeNO has a diagnostic value for bronchiolitis, but not for idiosyncratic disease.

## Availability of data and materials

The datasets used and/or analyzed during the present study are available from the corresponding author upon reasonable request.

## Ethics approval

All procedures performed in this study involving human participants were in accordance with the ethical standards of the institutional and/or national research committee and with the 1964 Helsinki Declaration and its later amendments or comparable ethical standards. All subjects were approved by Dongying People's Hospital.

## Authors’ contributions

XiaoYing Xu designed the research study. XiaoYing Xu, WeiNing Han and WeiPing Han performed the research. WeiNing Han and WeiPing Han provided help and advice on the experiments. WeiNing Han and WeiPing Han analyzed the data. XiaoYing Xu wrote the manuscript. XiaoYing Xu and WeiPing Han reviewed and edited the manuscript. All authors contributed to editorial changes in the manuscript. All authors read and approved the final manuscript.

## Funding

Not applicable.

## Conflicts of interest

The authors declare no conflicts of interest.
